# The Treatment of Whooping Cough.—II

**Published:** 1909-05-01

**Authors:** 


### Diseases of Children.

### THE TREATMENT OF WHOOPING COUGH.—II.

In a previous article we considered the general treatment of this affection and the local measures applicable to the nose and throat. The next group of remedies includes those which can be given by inhalation, either for the relief of the tracheo-laryngitis and secondary bronchitis or as local sedatives. One old-fashioned method was to send the child to gasworks to inhale the exhalations from the waste products. Whether these had any beneficial effect, or the improvement was due to the open air, is uncertain. Certainly it is inadvisable to use inhalations of coal gas, especially seeing that modern coal gas contains a high percentage of carbon monoxide. The chief drugs used for inhalation are compound tincture of benzoin; cresolin; creosote; benzol 1 in 10,000; iodide of ethyl; eucalyptus; the formalin lamp; terebene; thymol 1 in 5,000; and carbolic acid, of which 15-20 drops of a solution, 0.5 to 2.5 per cent. in strength, should be placed on cotton wool and inhaled for several hours daily. The latter may also be used as a spray, twice daily, at a distance of one yard from the child's head. The eyes should be protected by a bandage as it is liable to set up conjunctivitis. Some of these drugs can be dropped on a bib, constantly worn, so that the vapour is more or less continuously inhaled. Counter-irritants are of no value, except for secondary lung complications. A turpentine liniment may do good because the vapour is inhaled.

The number of drugs recommended for internal medication is very large; so great that there is little doubt the specific effect of any one of them is slight or absent. Belladonna still holds the first place in the minds of many. It reduces the number and severity of the spasms, checks the secretion of mucus, and is a cardiac and respiratory stimulant. It must be given in doses sufficiently large to produce erythematous flushing of the cheeks and dilatation of the pupils; ten minims of the tincture for each dose at six years of age, four minims at one year of age, every four hours. After a varying period it loses its effect. It can, with advantage, be combined with bromides in doses of 4-10 grains, as a sedative. Chloral hydrate is also a very useful sedative in small frequent doses, as long as the nutrition of the heart muscle is well maintained. Another valuable sedative is antipyrin in doses of one grain every four hours for each year of life, or half a grain if the child is under one year. It rarely causes rash in children, does not produce depression, stops the vomiting, and reduces the frequency of the paroxysms. Tussol, a compound of antipyrin, is given in similar doses. Other sedatives are heroin hydrochloride gr. $\frac{1}{30}$ to twice a day, codeine or morphine gr. $\frac{}{40}$ in one drachm of syrup of tolu three times a day at one year of age, butyl-chloral hydrate one grain every two to six hours (Eustace Smith), tincture of cannabis indica one to five minims every three hours, bromoform, fluoroform, cocaine, and inhalations of chloroform.

Experience has not proved that bromoform is any more efficacious than bromide and belladonna. On account of its high specific gravity it is liable to sink to the bottom of the bottle and be given in too large a dose. Several fatal cases have been due to this cause. The dose is from one to four minims three or four times a day, for each year of life. It can be given in pure form from a drop bottle, much the safest plan if there is a reliable nurse, or in emulsion or dissolved in alcohol. One drop of bromoform is soluble in an ounce of water, if five drops of alcohol are added. Poisonous doses produce narcosis, slow breathing, soft and intermittent pulse, an erythematous rash, and diminished corneal reflex. The treatment includes artificial respiration, mustard baths, faradisation, coffee, and alcohol.

A saturated solution of fluoroform in water, that is, 2.0-2.5 per cent. strength, in doses of a drachm to an ounce hourly, is said to reduce the duration of whooping to 6-18 days. For small infants one minim is given after each paroxysm and each dose is increased by one minim daily until 100 minims are taken in 24 hours. It is colourless, odourless, tasteless, apparently harmless, but rather expensive.

Quinine and euquinine are sometimes beneficial in large doses by mouth or rectum; gr. for each month of age under one year and $1\frac{1}{2}$ gr. for each year of age at 6 A.M., 2 P.M., and 10 P.M. Give it in full doses for three days and in half-doses for six days, and drop it gradually. Cocaine has been recommended as a local application to the external auditory meatus and the membrana tympani in 5-10 per cent. strength; as a spray to the fauces; and internally to stop vomiting: but probably grey powder and rhubarb are more effective and less harmful.

On the whole a prolonged experience of these various remedies has led the writer to rely on belladonna, bromides, antipyrin, chloral, and codeine, alone or in various combinations. It is important to remember that if there is much secondary catarrh the drugs and treatment appropriate for bronchitis are beneficial. Alkalies and iodides render the secretions less viscid and more easily got rid of, thus reducing the severity and duration of the paroxysms. Glycerine lozenges, etc., help to relieve the throat irritation.

But the essential point to bear in mind in the treatment of this disease is that two-thirds of the fatal cases are infants under one year of age, and about one-fourth of those under this age who get pertussis die. After the first year the mortality is only about 5 per cent. Constitutional debility, damp and cold weather, digestive troubles and bad hygiene are most dangerous accessories. Protect infants as far as possible from exposure. In estimating the value of treatment, and particularly of drugs, remember that mild cases get well in three or four weeks, even if untreated, and that more prolonged ones depend greatly on climatic conditions and environment, and persistent whooping is partly due to habit. There is no specific at present known, and probably a simple cough mixture, combined with general measures, is the best treatment.

